# A global database of woody tissue carbon concentrations

**DOI:** 10.1038/s41597-022-01396-1

**Published:** 2022-06-09

**Authors:** Mahendra Doraisami, Rosalyn Kish, Nicholas J. Paroshy, Grant M. Domke, Sean C. Thomas, Adam R. Martin

**Affiliations:** 1 Department of Physical and Environmental Sciences, University of Toronto Scarborough, M1C 1A4 Toronto, Canada; 2 USDA Forest Service, Northern Research Station, St. Paul, Minnesota USA; 3 Graduate Department of Forestry, Daniels Faculty of Architecture, Landscape, and Design, University of Toronto, Toronto, Ontario Canada

**Keywords:** Carbon cycle, Plant ecology, Carbon cycle, Forest ecology

## Abstract

Woody tissue carbon (C) concentration is a key wood trait necessary for accurately estimating forest C stocks and fluxes, which also varies widely across species and biomes. However, coarse approximations of woody tissue C (e.g., 50%) remain commonplace in forest C estimation and reporting protocols, despite leading to substantial errors in forest C estimates. Here, we describe the Global Woody Tissue Carbon Concentration Database (GLOWCAD): a database containing 3,676 individual records of woody tissue C concentrations from 864 tree species. Woody tissue C concentration data—i.e., the mass of C per unit dry mass—were obtained from live and dead woody tissues from 130 peer-reviewed sources published between 1980–2020. Auxiliary data for each observation include tissue type, as well as decay class and size characteristics for dead wood. In GLOWCAD, 1,242 data points are associated with geographic coordinates, and are therefore presented alongside 46 standardized bioclimatic variables extracted from climate databases. GLOWCAD represents the largest available woody tissue C concentration database, and informs studies on forest C estimation, as well as analyses evaluating the extent, causes, and consequences of inter- and intraspecific variation in wood chemical traits.

## Abstract (summary)

**Table:** 

Measurement(s)	wood carbon concentrations
Technology Type(s)	elemental analyzer
Factor Type(s)	species
Sample Characteristic - Organism	Plant
Sample Characteristic - Environment	terrestrial biome
Sample Characteristic - Location	Globe

## Background & Summary

Forests play a critical role in the global carbon (C) cycle, with the world’s forests storing an estimated 861 ± 66 Pg C across tropical (~471 Pg C), boreal (~272 Pg C), and temperate forest ecosystems (~119 Pg C)^1^. At the same time, C cycling in forested biomes is highly dynamic and transient, with estimates indicating that forests sequester between ~2.15 to 2.4 Pg C y^−1^ globally on average^1,2^. Throughout the 2000s, structurally intact old-growth forests accounted for ~0.85 Pg C y^−1^, while C sequestration was ~1.30 Pg C y^−1^ in secondary forests^2^. Tropical regions are particularly important in sequestering atmospheric carbon dioxide (CO_2_) in both regenerating^3–5^ and intact forests^1,6,7^. Nevertheless, recent analyses from both temperate^8^ and tropical regions^7^ have indicated that the magnitude of C sinks in old-growth forests are declining.

The amount of C stored within, and transferred to and from, trees and forests have been estimated from field- or remote-sensing-based observations of tree attributes, which are used to obtain estimates of tree- or forest aboveground biomass (AGB)^1,9–12^. Estimates of AGB are then converted into C estimates by multiplying these values by a woody tissue C concentration, commonly referred to in the literature as a C fraction^13–16^ (i.e., the mass of C per unit dry mass). Accurate woody tissue C concentration data are therefore critical in (1) accurately estimating terrestrial forest C budgets and sequestration rates^17^, (2) estimating the C emissions associated with land-use change^18^, and ultimately (3) informing decision-making related to the identification of forests with high C storage capacity^11^. Indeed, the Intergovernmental Panel on Climate Change’s (IPCC) Tier 3 C accounting protocols suggests that a “specific carbon fraction…should also be incorporated” when estimating C stocks and fluxes in AGB^13^. Moreover, woody tissue C concentration data can be employed in studies on the abiotic or biotic predictors of variation in – and possible adaptive significance of – wood chemical traits across tree species^19,20^, as well as evaluating the role that different sample extraction, preparation, and analytical methods have on wood C fractions^17^. Owing at least in part to a lack of large woody tissue C datasets, these research areas have received relatively little attention in comparison to other suites of plant traits^21^.

To date, most C estimation and reporting protocols use generic approximations of woody tissue C concentrations (namely, an assumption that 50% of AGB is comprised of C^13^), which has led to substantial systematic errors in forest C estimates. For example, our recent analyses indicated that generic woody tissue C fractions overestimate C stocks by approximately 8.9% in tropical forests^19^. Similar issues exist for the accounting of C stocks and fluxes in dead wood, with recent analyses indicating that generic dead wood C fractions may result in dead wood C pools being overestimated by ~3.0 Pg C globally^22^. Although multiple studies evaluating woody tissue C concentrations in trees globally through field- or meta-analyses now exist^19,23–25^, there is no single woody tissue C data repository to aid researchers in accessing and using these data.

To address these issues, we created and describe here the “Global Woody Tissue Carbon Concentration Database” (hereafter GLOWCAD^26^), which contains woody tissue C concentrations measured on live and dead tree tissues, spanning all forested biomes. By organizing and standardizing data from a range of taxonomic groups and woody tissue-types (described below), GLOWCAD represents a resource that helps improve our understanding of both global forest C dynamics and inter- and intraspecific variability in wood chemical traits. GLOWCAD only includes data from peer-reviewed sources. In addition to associated information on the taxonomic identities and woody tissue types for each woody tissue C data point, GLOWCAD includes geographical and associated bioclimatic data obtained from climate databases^27^.

**Fig. 1:**
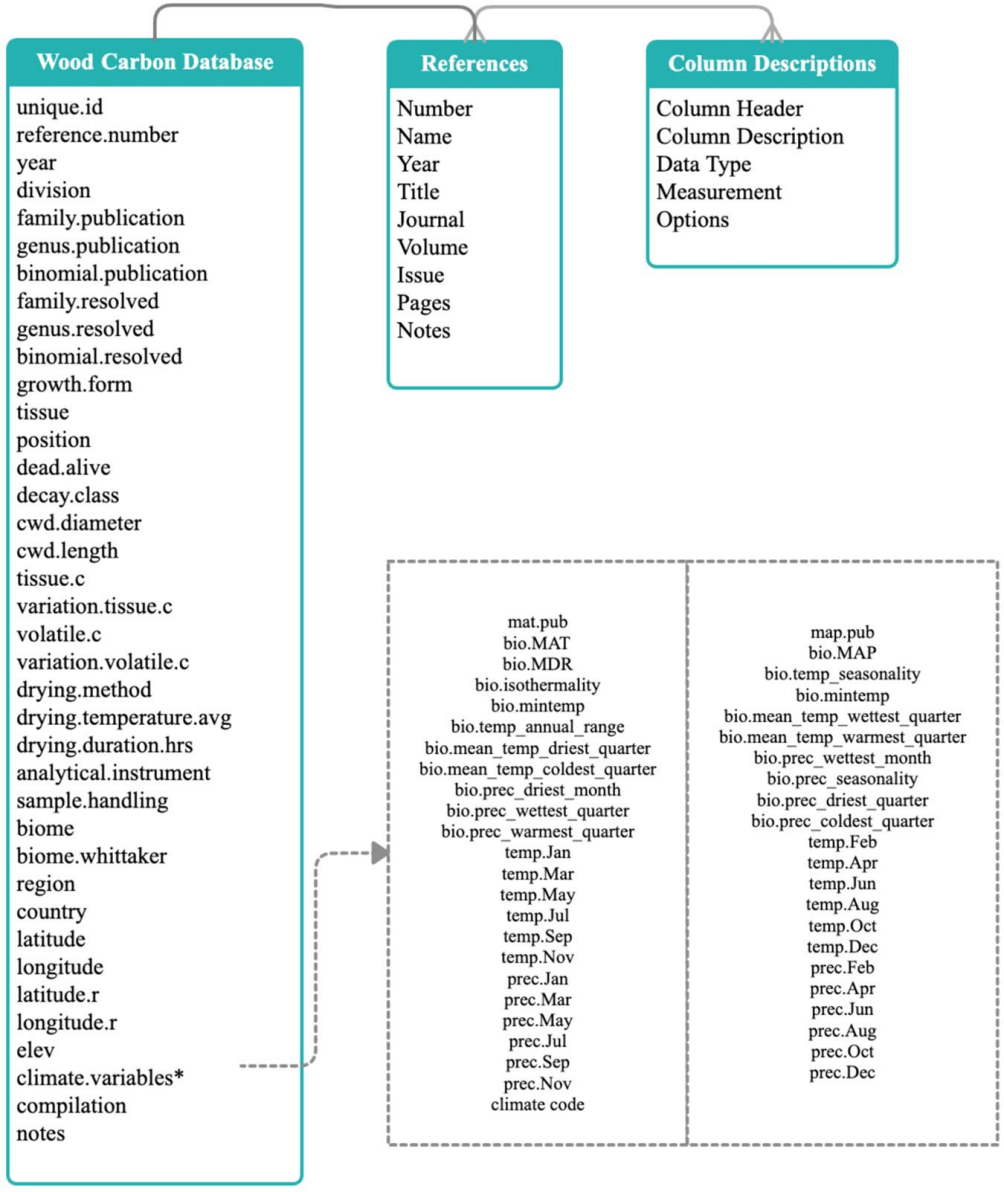
Structure of Global Woody Tissue Carbon Concentration Database (GLOWCAD). Teal boxes represent the three spreadsheets contained in GLOWCAD and include the column names of each record. Details for all measurements in the “Wood Carbon Database” worksheet are described in Supplementary Tables 1 and 2. Thick gray lines indicate links between worksheets, while gray dashed line indicates a sub-table containing sub-units of a primary variable.

Data records in GLOWCAD are stored in 3 easy-to-use Comma Separated Values (.csv) spreadsheets (Fig. 1). All spreadsheets comprise plain text, with the first spreadsheet (titled “Wood Carbon Database”) containing the core data (i.e., woody tissue C concentrations and related information), while the other spreadsheets provide descriptive supporting information including references (titled “References”) and column descriptions (titled “Column Descriptions”). GLOWCAD has been made publicly available through the Dryad Digital Repository, with existing applications including studies on: (1) woody tissue C concentrations variation across live trees^19,23,25^; (2) variation in dead woody tissue C concentrations^22^; (3) relationships between woody tissue C concentrations and tree life-history strategies^19,22^; and (4) climate correlates of woody tissue C concentrations in trees^28^.

## Methods

### Literature review

Data compilation expanded earlier versions of the GLOWCAD first initiated in 2012^25^, and more recently published in 2018^19^ and 2021^29^. GLOWCAD is therefore based on a systematic search on primary literature of all peer-reviewed papers that cited previously published studies on woody tissue C concentrations^19,23–25^. We searched key terms “carbon”, “tree”, “wood carbon”, “coarse woody debris”, “dead wood”, and “wood nutrient”, as well as “carbon” alongside major tree tissue types (including “wood”, “bark”, “root” and “stem”), within four web-based platforms (Google Scholar, Web of Science, Web of Knowledge, and Scopus), in order to identify peer-reviewed publications that present species- specific woody tissue C concentration data.

In addition to peer-reviewed papers, other sources of data included in GLOWCAD include the TRY Plant Trait Database (v. 5.0)^29^ and the Global Root Traits (GRooT) Database^28^. The TRY datasets included are the Subarctic Plant Species Database (dataset ID 105), Plant Traits for Pinus and Juniperus Forests in Arizona (dataset ID 193), Plant Physiology Database (dataset ID 97), Panama Tree Traits Database (dataset ID 230), FRED database (dataset ID 339), and the ECOCRAFT Database (dataset ID 12). While most root C values in GRooT were included in the FRED database (v. 2.0)^30^, data from 10 papers in GRooT were added to GLOWCAD (*n* = 197 data points): Isaac *et al*.^31^; Liu *et al*.^32^; Wang *et al*.^33^; Minden *et al*.^34^; Alameda *et al*.^35^; Aubin *et al*.^36^; Fernández-García *et al*.^37^; Grechi *et al*.^38^; Ineson *et al*.^39^; and Pregitzer *et al*.^40^.

### Wood C data attributes

To be included in GLOWCAD, the species-specific binomial nomenclature and tissue-specific information for each woody tissue C sample was required. A detailed field and lab methodology was also necessary, in order to maximize our sample size while permitting reliable species- and tissue-specific analysis. Where a single paper contained multiple tissue- and species-specific woody tissue C records, all the published values were recorded. In the majority of cases, woody tissue C data were extracted directly from published tables or from supplementary data of the articles. In instances where woody tissue C data were published as figures, the data was extracted using the WebPlotDigitizer v4.2 software^41^. If species-by-tissue-specific woody tissue C data were not published, the corresponding authors were contacted to provide data.

Each published woody tissue C record was then classified according to the forest biome in which it was sampled. A small number of studies (e.g.^42^) presented both boreal and temperate data, which were differentiated in our database based on the sampling location coupled with a consultation of species distribution maps. Species taxonomy was first recorded as presented as in published articles. A final list of taxa was then compared with, and resolved according to, the Taxonomic Name Resolution Service v. 4.0^43^. Both original and resolved taxonomy is maintained in GLOWCAD. Inclusion of new published data was halted as of Dec. 31, 2020.

### Dead wood C data attributes

When classifying dead wood data, we considered three primary factors associated with woody tissue decomposition and related chemical change: A) decay class (DC), B) position, and C) size (diameter and length). In the majority of publications, dead woody tissue C values were reported along a conventional 1–5 DC scale. These values were included in GLOWCAD as published, while noting the DC scale employed. In cases where DC was reported as a two-category range (e.g. DC 1–2), the higher DC was included in GLOWCAD. In cases where a multiple category DC was presented (e.g. DC 3–5), the middle DC value was used in GLOWCAD. In the few instances DC was reported along a 0–5 point scale (where DC of 0 was defined as dead and not live wood), dead wood reported with a DC of 0 was classified as DC 1. Lastly, in a subset of papers the number of years since tree death (instead of DC) was reported. In these cases, years since death were converted to DC based on published decay class transition metrics (e.g.^44^). When classifying position of dead wood, “standing” referred to snags and suspended woody debris, and “downed” referred to anything sampled from the forest floor. The default position was “downed” for the few publications that did not specify position.

### GLOWCAD structure

The structure of GLOWCAD is simple to navigate (Fig. 1). Within GLOWCAD, all the woody tissue C data is present under the “Wood Carbon Database” spreadsheet. In this spreadsheet, a unique number (i.e., ‘unique.id’) of all woody tissue C data is specified beside the reference from which it was obtained. The value of the ‘reference.number’ corresponds to the detailed citation presented in the “References” spreadsheet, which links the ‘reference.number’ with the author(s)’ name and publication year, title, journal, volume, issue, and pages.

When inputting woody tissue C data from publications into GLOWCAD, the latitude and longitude were also recorded in the database when explicitly stated in the original publication. General climate information such as mean annual temperature (MAT) and mean annual precipitation (MAP) of the study region were recorded as an average. The study regions’ latitude and longitude were also used to further describe its climate with WorldClim (v.2) data^27^. However, when a range of geographic coordinates or a map was provided, climate data were not generated from these since averages MAT and MAP may be imprecise. We used MAT and MAP obtained from WorldClim (v.2) to label the study region’s dominant Whittaker biome^45^, and therefore categorize the region as one of Boreal forest, Subtropical desert, Temperate grassland/desert, Temperate rain forest, Temperate seasonal forest, Tropical rain forest, Tropical seasonal forest/savanna, or Woodland/shrubland. A list containing the details collected from each publication is presented (Supplementary Table 1). Bioclimatic variables and other climate data associated with each study location were retrieved from WorldClim (v.2)^27^ and added alongside woody tissue C data (Supplementary Table 2).

### Previous versions of GLOWCAD

GLOWCAD is the fourth iteration of the woody tissue C dataset, though these earlier versions did not use the same acronym, and contained differing sets/ subsets of data based on different research questions. Three earlier versions are publicly hosted in the TRY Plant Trait Database, such that: 1) the first version contained *n* = 973 observations of dead wood C only, from 121 species; 2) the second version contained *n* = 1,145 observations of live woody tissue C only, from 415 species paired with geographic coordinates and climate data; and 3) the third version contained *n* = 2,432 observations of live woody tissue C only, from 636 species including all of the observations of the previous version .

GLOWCAD is a single data product which consolidates the dead and live woody tissue C observations of all prior iterations (where *n* = 3,405), and includes 271 new woody tissue C observations from 10 additional publications^31–40,46^. In sum, *n* = 3,676 data points in the GLOWCAD version described here. Unlike previous versions, GLOWCAD also includes information on growth habit or ‘woodiness’ (described below) and the original binomial nomenclatures as listed in their publications.

## Data Records

**Fig. 2:**
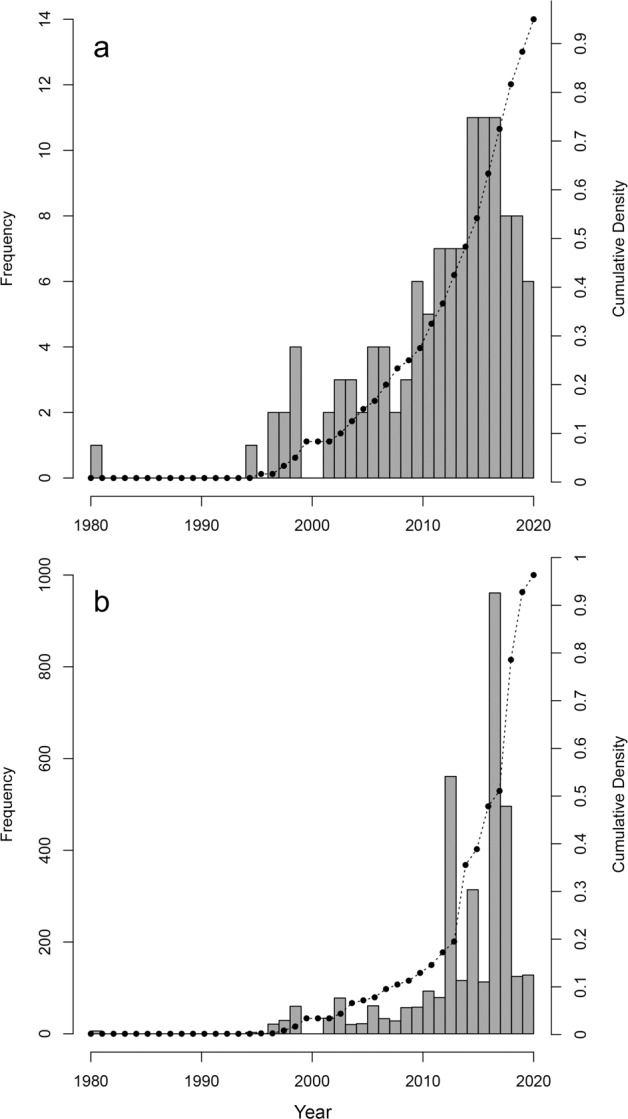
Number of peer-reviewed publications (Panel (**a**)) and observations (Panel (**b**)) included in Global Woody Tissue Carbon Concentration Database (GLOWCAD) across publication year. Gray bars indicate the number of publications or woody tissue carbon values included in GLOWCAD (corresponding to the y-axis), while black circles and dotted lines correspond to a cumulative probability density function (corresponding to the z-axis).

**Fig. 3:**
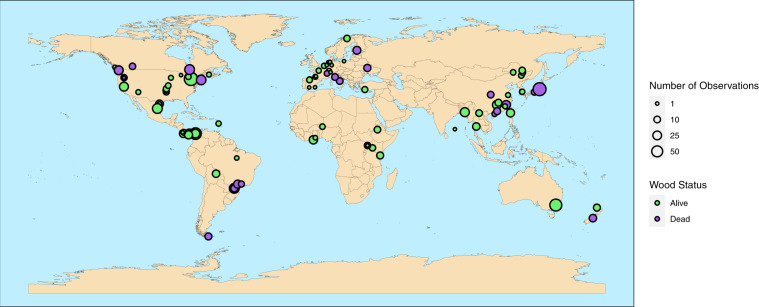
Woody tissue carbon concentration sampling sites for data sources included in the Global Woody Tissue Carbon Concentration Database (GLOWCAD). Data point colours correspond to tree status, where dead woody tissue is represented in green and live woody tissue is represented in purple. Point sizes are proportional to the number of woody tissue C observations recorded at a site on a continuous scale, ranging from 1–85 observations.

GLOWCAD is stored in .csv format at the Dryad Digital Repository (10.5061/dryad.18931zcxk). Data outputs consist of a single database of 3,676 woody tissue C observations from 130 sources^17,23,24,30–40,42,44,46–159^ published between 1980 and 2020 (Fig. 2), which includes C concentrations of woody tissues from 864 tree/shrub species sampled across all continents except Antarctica (Fig. 3). While data exists from papers published since 1980, the large majority (86%) of the data in GLOWCAD (*n* = 3,154 data points) is derived from sources published in or after 2010 (Fig. 1).

**Fig. 4:**
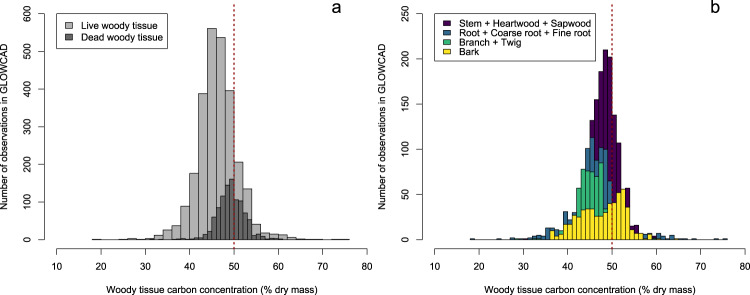
Variation in woody tissue C concentrations (% dry mass) in GLOWCAD. Panel (**a**) compares the distribution of C concentrations between live woody tissues (light grey bars) and dead woody tissues (dark grey bars). Panel (**b**) compares the distribution of C concentrations among woody tissue types: stem, heartwood and sapwood (purple bars); root, coarse root and fine root (blue bars); branch and twig (green bars); and bark (yellow bars).

GLOWCAD includes woody tissue C values from 414 genera and 107 families, with the Pinaceae (*n* = 927 data points), Fabaceae (*n* = 383 data points), Fagaceae (*n* = 335 data points), Cupressaceae (*n* = 159 data points) and Betulaceae (*n* = 146 data points) being most well represented (Supplementary Tables 3–5). Across biomes, most woody tissue C data in GLOWCAD are derived from tropical forests (*n* = 1,513 data points), followed by temperate (*n* = 1,202 data points), subtropical/ Mediterranean (*n* = 518 data points) and boreal (*n* = 301 data points) forests. Across the entire database, woody tissue C ranged from 18.4–75.1% (Fig. 4).

In GLOWCAD, 73% of data points were obtained from woody tissue measurements of live plants (*n = *2,671 data points), while the remaining 27% (*n* = 1,005 data points) came from dead plant measurements. In regard to tissue types, stems (inclusive of heartwood and sapwood; *n* = 1,523 data points), roots (inclusive of fine-root and coarse-root; *n* = 986 data points) and branches (inclusive of both large and small branches/twigs; *n* = 619 data points) were most well represented (Table 1; Fig. 4). Additionally, woody tissue C data were retrieved from publications spanning a wide climatic range, with a MAT ranging from −5.4–29 °C (across *n* = 1,326 data points), and MAP ranging from 160–5,130 mm (*n* = 1,455 data points).

**Table 1:** Sample sizes and ranges of woody tissue carbon concentration data in the Global Woody Tissue Carbon Concentration Database (GLOWCAD).

Tissue type	Live woody tissue			Dead woody tissue		
	*n*	Minimum	Maximum	*n*	Minimum	Maximum
Bark	319	36.0	65.0	229	41.0	59.0
Branch	337	28.0	59.2	85	41.5	53.8
Coarse root	345	28.0	58.3	0	NA	NA
Fine root	545	29.8	75.12	0	NA	NA
Heartwood	28	47.1	55.1	0	NA	NA
Root	39	18.4	51	57	44.5	50.0
Sapwood	33	47.1	54.1	0	NA	NA
Stem	828	30.5	60.68	634	29.4	60.2
Twig	197	33.0	54.9	0	NA	NA

The foremost drying method employed by publications incorporated into GLOWCAD was conventional oven-drying (*n* = 1,941 data points), while the least common was the Minimizing the Loss of Carbon (MLC) method described by Jones and O’Hara (2016^96^; *n* = 9 data points). Drying temperatures ranged widely from 18–110 °C, with drying durations spanning 5–360 hours. The majority of publications made use of Elemental Analyzers (corresponding to *n* = 2,760 data points) when estimating woody tissue C concentrations. In sum, 34% of observations in GLOWCAD (*n* = 1,241 data points) were associated with exact geographic coordinates of their sampling locations (i.e, not a range of latitude and longitude), and only these observations were assigned climate information from WorldClim (v.2)^27^.

## Technical Validation

### Trait data validation

All 3,676 records included in GLOWCAD were obtained from peer-reviewed scientific journals, or indirectly, through the TRY Functional Trait Database or Global Root Traits Database. Each specific record is linked to its original reference, allowing users to verify and validate the accuracy of tissue C data and data source. All data in GLOWCAD was thoroughly screened to ensure accuracy, and appropriate methods of data acquisition. Specifically, woody tissue C values had to be measured directly, and not approximated based on secondary sources. Data that did not meet these criteria were excluded from GLOWCAD.

### Taxonomic validation

**Fig. 5:**
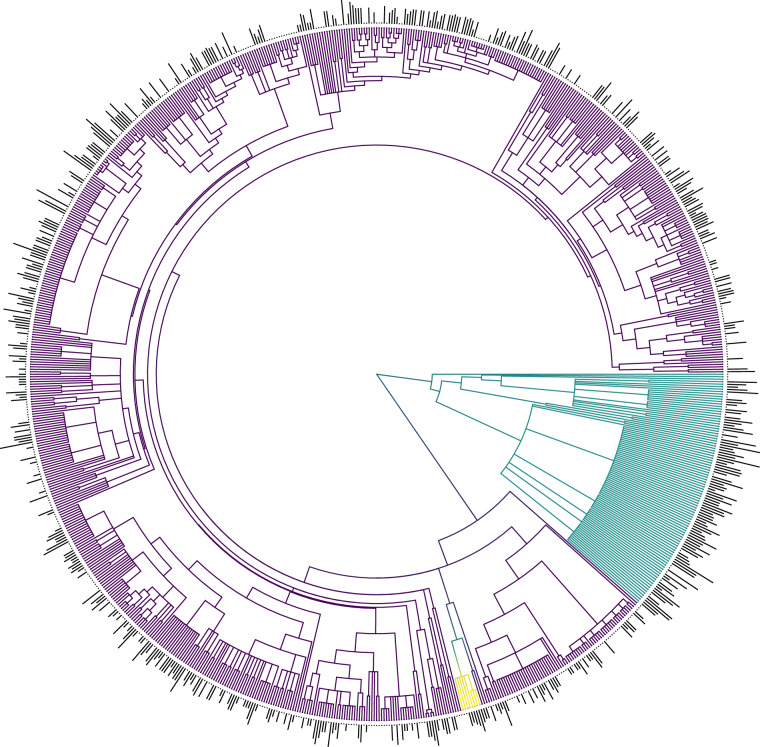
Phylogenetic coverage of species represented within the Global Woody Tissue Carbon Concentration Database (GLOWCAD). Colours mapped onto the phylogenetic tree correspond to 1) two major plant clades including gymnosperms (in blue, *n* = 100 species), angiosperms (in purple, *n* = 772 species), and 2) angiosperm ‘palm’ family, Arecaceae (in yellow, *n* = 8 species). Each branch represents a species in GLOWCAD (*n* = 864 total). Bars on the outer ring depict the sample sizes for each species (number of observations proportional to the logarithm of base 10), which are presented in full in Supplementary Tables 3–5. Phylogeny here is based on the Angiosperm Phylogeny Group megatree (R2012089.new) with branch lengths corresponding to clade ages based on fossil records^165,166^.

Across the 40-year period during which data was collected (Fig. 1), tree species may have been misidentified or had their taxonomic information updated. To address these discrepancies and ensure that the most up-to-date taxonomic information is included in GLOWCAD, taxonomic information was directly recorded from original papers, and then verified and adjusted accordingly to reflect the appropriate name listed in the Taxonomic Name Resolution Service v. 4.0^33^. All woody tissue C records included binomial nomenclature, and records without this degree of specificity were omitted from GLOWCAD. Phylogenetic coverage associated with the resolved taxonomy within GLOWCAD are presented in Fig. 5.

### Growth habit validation

Growth habit or ‘woodiness’ was evaluated for all species included in GLOWCAD to ensure that woody tissue C data corresponded only to woody plant species, based on a functional definition of “woody”: i.e., having a persistent aboveground stem^160^. Therefore, all species were cross-referenced with those included in a global growth habit dataset^160^ and growth habits – defined here as trees, shrubs, or shrub/tree– were assigned. Species of the Arecaceae (palm) family (*n* = 8 species) were also included in GLOWCAD (*n* = 32 data points) since these 1) met the functional definition of “wood” and are 2) are important contributors to aboveground biomass C in Neotropical forests, relative to other biogeographic locations^161^, monocot species^162^ and non-conventional woody species (e.g. tree ferns)^163^.

### Climate data validation

Bioclimatic variables were assigned to observations which were accompanied by the specific geographic coordinates (excluding ranges) of their sample location, using WorldClim (v.2). In GLOWCAD 34% of woody tissue C observations include a WorldClim-derived estimate of MAT and MAP (*n* = 1,241 data points). Linear regression models indicated that statistically significant positive relationships existed between (1) publication- vs. WorldClim-derived estimates of MAT (*p*<0.001, *r*^2^ = 0.95, model slope = 0.97 ± 0.01 (s.e.)) and (2) publication- vs. WorldClim-derived estimates of MAP (*p*<0.001, *r*^2^ = 0.82, model slope = 1.23 ± 0.02 (s.e.)).

## Usage Notes

GLOWCAD is openly available for use in any application. It can be accessed via (1) the DRYAD Digital Repository (10.5061/dryad.18931zcxk), (2) a GitHub repository, (3) the TRY Plant Trait Database, and (4) upon request to the corresponding author. GLOWCAD is licensed under CC-BY 4.0.

## Supplementary information

Supplementary Table 1

Supplementary Table 2

Supplementary Table 3

Supplementary Table 4

Supplementary Table 5

## Data Availability

All analyses used to generate figures and summary statistics were performed in R (v.4.1.2)^164^. No custom computer code or algorithms were used to generate the data presented in the manuscript.
